# Mendelian randomization analysis of factors related to ovulation and reproductive function and endometrial cancer risk

**DOI:** 10.1186/s12916-022-02585-w

**Published:** 2022-11-01

**Authors:** Shannon D’Urso, Pooja Arumugam, Therese Weider, Liang-Dar Hwang, Tom A. Bond, John P. Kemp, Nicole M. Warrington, David M. Evans, Tracy A. O’Mara, Gunn-Helen Moen

**Affiliations:** 1 Institute for Molecular Bioscience, The University of Queensland, Brisbane, Australia; 2 School of Biomedical Sciences, Faculty of Medicine, The University of Queensland, Brisbane, Australia; 3 Institute of Clinical Medicine, Faculty of Medicine, University of Oslo, Oslo, Norway; 4 Department of Endocrinology, Morbid Obesity and Preventive Medicine, Oslo University Hospital, Oslo, Norway; 5 MRC Integrative Epidemiology Unit at the University of Bristol, Bristol, UK; 6 The University of Queensland Diamantina Institute, The University of Queensland, Brisbane, QLD Australia; 7 Bristol Medical School, Population Health Science, University of Bristol, Bristol, UK; 8 Department of Epidemiology and Biostatistics, Imperial College London, London, UK; 9 K.G. Jebsen Center for Genetic Epidemiology, Department of Public Health and Nursing, NTNU, Norwegian University of Science and Technology, Trondheim, Norway; 10 Cancer Research Program, QIMR Berghofer Medical Research Institute, Brisbane, QLD Australia

**Keywords:** Mendelian randomization, Endometrial cancer, Fertility, UK biobank, GWAS, Years ovulating, Number of live births, Age at last live birth

## Abstract

**Background:**

Observational epidemiological studies suggest a link between several factors related to ovulation and reproductive function and endometrial cancer (EC) risk; however, it is not clear whether these relationships are causal, and whether the risk factors act independently of each other. The aim of this study was to investigate putative causal relationships between the number of live births, age at last live birth, and years ovulating and EC risk.

**Methods:**

We conducted a series of observational analyses to investigate various risk factors and EC risk in the UK Biobank (UKBB). Additionally, multivariate analysis was performed to elucidate the relationship between the number of live births, age at last live birth, and years ovulating and other related factors such as age at natural menopause, age at menarche, and body mass index (BMI). Secondly, we used Mendelian randomization (MR) to assess if these observed relationships were causal. Genome-wide significant single nucleotide polymorphisms (SNPs) were extracted from previous studies of woman’s number of live births, age at menopause and menarche, and BMI. We conducted a genome-wide association analysis using the UKBB to identify SNPs associated with years ovulating, years using the contraceptive pill, and age at last live birth.

**Results:**

We found evidence for a causal effect of the number of live births (inverse variance weighted (IVW) odds ratio (OR): 0.537, *p* = 0.006), the number of years ovulating (IVW OR: 1.051, *p* = 0.014), in addition to the known risk factors BMI, age at menarche, and age at menopause on EC risk in the univariate MR analyses. Due to the close relationships between these factors, we followed up with multivariable MR (MVMR) analysis. Results from the MVMR analysis showed that number of live births had a causal effect on EC risk (OR: 0.783, *p* = 0.036) independent of BMI, age at menarche and age at menopause.

**Conclusions:**

MVMR analysis showed that the number of live births causally reduced the risk of EC.

**Supplementary Information:**

The online version contains supplementary material available at 10.1186/s12916-022-02585-w.

## Background

Endometrial cancer (EC) is the sixth most commonly diagnosed cancer in women worldwide and is increasing in incidence across the world, particularly in developing nations [1, 2]. A number of observational studies have shown a relationship between different factors and EC risk. In particular, late menarche, early menopause, and the use of some forms of oral contraceptives are observationally associated with decreased risk of EC (for an in-depth review, see Webb (2015) [3]). One potential explanation for these associations is that higher lifetime estrogen exposure causally increases EC risk [4, 5]. This could explain why a short reproductive span (i.e., late menarche and early menopause) is associated with protection against EC in observational epidemiological studies [3–5].

Oral estrogen is known to promote endometrial cell proliferation and could favor tumorigenesis [6, 7]. Hormone replacement therapy (HRT) with estrogen only, used to ameliorate menopausal symptoms, is associated with an increased EC risk [8] and Mendelian randomization (MR) analysis has shown a causal link between estradiol and EC [9]. Progesterone, on the other hand, downregulates estrogen receptors in the endometrium and promotes cell differentiation, thus opposing the mitogenic effects of estrogen [10, 11]. HRT consisting of estrogen in combination with progestins reduces EC risk compared to estrogen therapy alone [12] and supports an unopposed estrogen theory of EC carcinogenesis. It may therefore not be estrogen itself that is causal for the development of EC, but the estrogen/progesterone ratio. The combination type of oral contraceptives, which contain both estrogen and synthetic progesterone (progestin), is associated with decreased EC risk [13], and the protective effect is thought to be cumulative [14]. Unfortunately, studies of progestin-only contraceptives are scarce, possibly since progestin-only birth control is newer and less commonly used. Nevertheless, current research into administering progestins via an intrauterine device to treat certain subtypes of EC [15] has given further support to the hypothesis that progesterone might be protective in terms of EC incidence.

Another factor observationally associated with reduced risk of EC is the total number of pregnancies [16]. This phenomenon has been hypothesized as being the result of the shedding of malignant and premalignant endometrial cells during and after childbirth and also potentially from exposure to high levels of progesterone in late stages of pregnancy [17–19]. More recent work has found a protective effect (although to a lesser extent) of incomplete pregnancies on EC risk (i.e., spontaneous and induced abortions [20]). The protective effect associated with both complete and incomplete pregnancies is greater than that of oral contraceptive use [20]. Additionally, age at last live birth has been associated with decreased EC risk [21]. The reason behind this protective association is unknown, but it has been hypothesized that since older women have a higher risk of malignant and premalignant endometrial cells, removal of these cells (either mechanically through birth or biochemically via high progesterone levels, as shown in trials using progesterone as EC treatment [15, 22]) may be more beneficial with increasing age [18].

In addition to these abovementioned factors, obesity is the risk factor that has the strongest association with EC risk [23, 24]. It is estimated to account for approximately 40% of EC incidence in developed countries [25] and may be one reason behind increasing rates of malignancy in rapidly developing nations. The association between obesity and EC risk is also believed to be an effect of estrogen exposure, due to increased conversion of androgenic precursors to estradiol in adipose tissue [26].

Much of the research in the area of factors related to ovulation and reproductive function leading to EC risk has been limited to observational epidemiological studies. Therefore, conclusions regarding causality cannot easily be drawn from these studies due to the possibility of bias and latent confounding. MR is an epidemiological method that uses genetic variants as instrumental variables to investigate whether an observational association between an exposure and an outcome represents a causal relationship [27–30], and is robust to some of the limitations of observational epidemiological studies. Body mass index (BMI) is one such risk factor that has further been studied using MR and has been reported in several papers to causally increase the risk of EC [31–33]. One of which shows that fasting insulin, bioavailable testosterone, and sex hormone-binding globulin seem to mediate the relationship between BMI and EC risk [33].

The aim of this current study was to investigate putative causal relationships between the number of live births, age at last live birth, and years ovulating and EC risk, by conducting observational analysis and univariate MR analysis. Additionally multivariate analysis was performed to elucidate the relationship between these primary exposures and other related factors such as age at natural menopause, age at menarche, and BMI.

## Methods

Detailed description of the methods is provided below. As an overview, firstly, we conducted a series of observational epidemiological analysis using the UK Biobank (UKBB) [34, 35]. Secondly, we conducted univariate MR analyses between (1) BMI; (2) factors related to ovulatory function (years ovulating, age at menarche, and age at natural menopause); (3) factors related to reproductive function (number of live birth, age at last live birth); and (4) oral contraceptive pill use on EC risk using publicly available genome-wide association study (GWAS) summary statistics and the UKBB [34, 35]. However, a complication of such analyses is that many single nucleotide polymorphisms (SNPs) proxy not just one factor but show pleiotropic associations with several phenotypes, often referred to as horizontal pleiotropy. Naïve use of univariate MR methods in these situations can produce biased estimates of the causal effect if this horizontal pleiotropy is not accounted for. For this reason, we also used multivariable MR (MVMR) [36] to estimate the direct causal effects of our main exposures (number of live births, age at last live birth, and years ovulating) that we found to be significant in the univariate MR analyses, on the risk of EC, conditional on other known risk factors (BMI, age at menarche, and age at natural menopause). MVMR accounts for any horizontal pleiotropy that influences the outcome through the multiple exposure variables that form part of the statistical model. Application of MVMR can thus help disentangle the relationship between genetically correlated exposures and obtain consistent and direct causal effect estimates of each exposure on the outcome of interest [36].

### Observational analysis in UKBB

#### Cohort description

The UKBB is a large prospective population based cohort containing ~ 500,000 individuals (approximately 273,000 women), with a variety of phenotypic and genome-wide genetic data available [35]. We used the UKBB for our observational, GWAS, and MR analyses.

The UKBB has ethical approval from the North West Multi-Centre Research Ethics Committee (MREC), which covers the UK, and all participants provided written informed consent.

#### Genetic data

We utilized imputed genetic data from the October 2019 (version 3) release of the UKBB for our analyses (Application ID: 53,641). In addition to the quality control metrics performed centrally by the UKBB [34], we defined a subset of unrelated “white European” women. We excluded those with putative sex chromosome aneuploidy, high heterozygosity or missing rate, or a mismatch between submitted and inferred sex as identified by the UKBB (total *N* = 1932). We excluded women who we did not identify as ancestrally European using *K*-means clustering applied to the first four genetic principal components generated from the 1000 Genomes Project [38]. We also excluded women who had withdrawn their consent to participate in the study as of February 2021. A total of 251,058 women of white European ancestry were available for further analyses.

#### Phenotypes

**Fig. 1:**
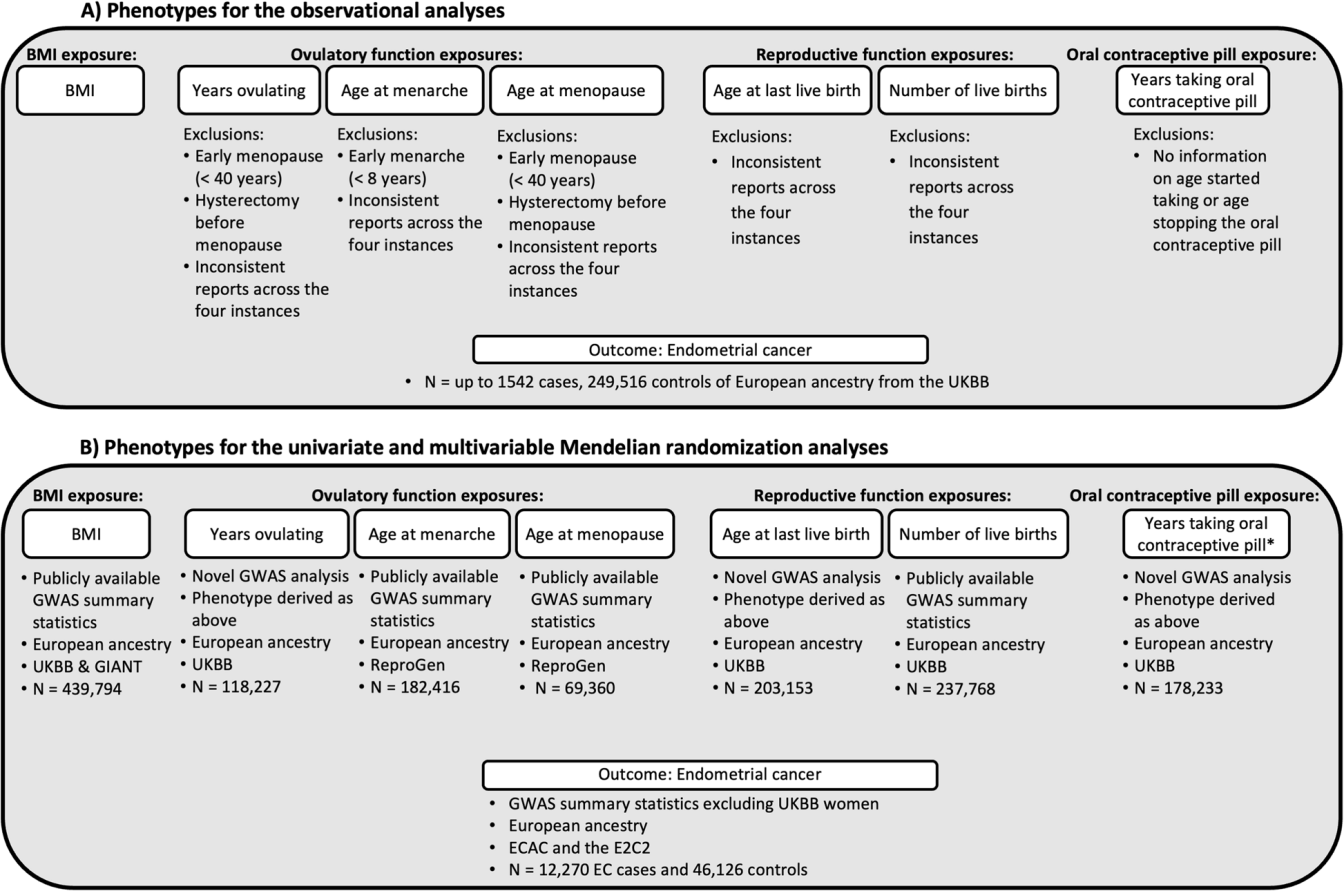
Data overview, quality control, and phenotype selection. Panel **A** shows the main phenotypes investigated in the observational analyses, where exposure variables and the outcome (EC) were derived from UKBB data. Panel **B** shows the main phenotypes investigated in the univariate and multivariable MR analyses. * indicates that we were unable to perform MR analyses on the years taking oral contraceptive pill phenotype as no genome-wide significant variants were identified in the GWAS. EC, endometrial cancer; ECAC, Endometrial Cancer Association Consortium; E2C2, Epidemiology of Endometrial Cancer Consortium; GWAS, genome-wide association study; MR, Mendelian randomization; UKBB, UK Biobank

A detailed description of the phenotype derivations for (1) BMI; (2) factors related to ovulatory function (years of ovulation, age at menarche, and age at natural menopause); (3) factors related to reproductive function (number of live birth, age at last live birth); and (4) oral contraceptive pill use will follow in subsequent paragraphs, with an overview of data and data quality control shown in Fig. 1.

##### Endometrial cancer

Women with EC were defined by those with an ICD-10 code C54.1 (*N* = 1542) in the national cancer registry. We used C54.1 as this is referred to as malignant neoplasm of corpus uteri in the endometrium, to ensure only endometrial carcinoma is included in the analysis. Women with either no cancer or a different type of cancer, were used as controls.

##### BMI

BMI, constructed from height and weight measures taken at the initial assessment center visit, were extracted. BMI measures were available for up to 268,277 women and range from 12 to 75 kg/m^2^.

##### Factors related to ovulatory function

Age at menarche and age at natural menopause are self-reported in the UKBB as the age of the first and last menstrual period respectively, ranging from 5 to 25 for age at menarche, and 18 to 68 for age at menopause. Years ovulating was defined as time between self-reported age at menarche and menopause, after accounting for years of oral contraceptive pill use and the number of full-term pregnancies (sum of live and stillbirths) in post-menopausal UKBB women. The formula below was adapted from previous literature [5] based on available variables in the UKBB [4, 5]:

$$\mathrm{Years \ ovulating }=\mathrm{ years \ menstruating }-\mathrm{ years \ on \ pill }- 0.75 \times (\mathrm{live \ births }+\mathrm{ stillbirths})$$

Women with an early age at menarche of < 8 years were excluded from this analysis (*N* = 15). In addition, women with a history of hysterectomy before menopause (*N* = 12,539), who were not sure of their age at menopause due to their hysterectomy (*N* = 30,788), or early menopause (< 40 years; *N* = 3,144) were also excluded. For menopause and menarche, we used the average age reported across multiple reporting instances, and women who differed in their reported ages by greater than 3 years were excluded from the analysis. 118,227 women were included in the years of ovulating analyses.

##### Factors related to reproductive function

The number of live births is self-reported in the UKBB and ranges from 0 to 22, 0 to 14 for the number of stillbirths, 0 to 21 for the number of spontaneous miscarriages, and 0 to 22 for the number of pregnancy terminations. Women with any inconsistencies in their reported numbers across four measurement occasions were excluded (*N* = 8). The number of pregnancies was calculated as the sum of live births, stillbirths, spontaneous miscarriages, and terminations. Women who had never had a live or stillbirth, miscarriage, or termination were included in the analysis with the value 0, and women with a medical history of hysterectomy before menopause or early menopause (< 40 years) were excluded.

Age at last live birth was defined as the age of primiparous women at birth (for women who reported only one live birth) or the age at last live birth as reported by women who reported multiple live births. Women with inconsistent reports of age at last live birth across the four data collection instances were excluded (*N* = 263). 220,419 women had information available for their age at last live birth.

##### Oral contraceptive pill

Years taking the oral contraceptive pill for UKBB women was calculated as the difference between self-reported age when women started using the oral contraceptive pill, and the age when they last used the oral contraceptive pill (for women who reported ever taking the oral contraceptive pill). 178,233 women had information available to derive the years taking the oral contraceptive pill phenotype. Ever taken the contraceptive pill is reported as a “yes” or “no” variable in the UKBB.

##### Confounding variables

The schooling qualifications variable in the UKBB involves six categories of educational attainment (college or university degree, A levels/advanced subsidiary levels or equivalent, O levels/general certificates of secondary education or equivalent, certificates of secondary education or equivalent, national vocational qualifications or higher national diplomas or higher national certificates or equivalent, other professional qualifications, e.g., nursing, teaching). The Townsend Deprivation Index is reported in the UKBB as a score based on participant postcodes, ranging from − 6.26 (lower deprivation) to 11 (higher deprivation).

#### Observational epidemiological analyses

We performed univariate logistic regression analysis to assess the observational association between several factors and EC risk in women of European ancestry in the UKBB. The phenotypes investigated included the number of live births, stillbirths, pregnancy terminations, miscarriages, total number of pregnancies (defined as live births + stillbirths + terminations + miscarriages), age at last live birth, ever taken the oral contraceptive pill, the number of years on the oral contraceptive pill, age at menopause, age at menarche, years ovulating and BMI. Logistic regression analyses were also performed including two potential confounders, Townsend Deprivation Index and educational attainment (measured by schooling qualifications), and EC risk.

We also conducted a series of multivariate observational analyses for EC risk. The first model included EC and all of the exposures investigated in the MVMR (see below; number of live births, age at last live birth, age at menarche, age at menopause, BMI) as well as educational attainment, due to the strong signal observed in the univariate observational analysis. The second model further explored the effect of the number of terminations on EC risk, due to the strong association observed in the univariate observational analyses, while adjusting for other factors (number of live births, age at last live birth, age at menarche, age at menopause, BMI) and educational attainment.

We conducted further sensitivity analyses to investigate the observational and potentially causal relationships between the number of live births and EC risk. While univariate observational analyses found the number of live births to be significantly associated with EC risk, the association attenuated in the multivariate observational analyses. Further regression analysis was performed, where we investigated the number of live births and EC risk, while adjusting for a single other risk factors (i.e., adjusting for one of age at menarche, age at menopause, the number of miscarriages, the number of terminations, BMI, educational attainment, ever taken the oral contraceptive pill, and age at last live birth).

All analyses were performed in R version 3.4.3.

### Genome-wide association analysis in UKBB

We conducted GWAS of years of ovulating information, age at last live birth, and years of taking the contraceptive pill in the women from the UKBB, to obtain instruments for MR analyses, as there are no previously published GWASs of these traits which identify robustly associated SNPs. We used fastGWA [39] in the Genome-wide Complex Trait Analysis (GCTA) software (v1.93.2beta) [40] to conduct the analyses, which utilizes a linear mixed model to account for population stratification and cryptic relatedness. A genetic relationship matrix (GRM) was generated from cleaned called genotype data (excluding variants with a Minor allele frequency (MAF) < 0.01, genotyping rate < 10%, and failed Hardy–Weinberg equilibrium exact test *p* < 1 × 10^−6^) and converted to a sparse GRM (elements < 0.05 were set to zero) before being included in the mixed linear model-based GWAS analyses. The model parameters were estimated once using SNPs across all chromosomes, then loaded before running association analysis for each chromosome separately. Imputed variants with an INFO score > 0.8, MAF > 0.0001, and missingness rate < 0.10 were used for the GWAS analyses, resulting in a total of *N* = 18,557,407 SNPs included. Covariates included UKBB assessment center, genotyping batch, year of birth, and the top ten genetic principal components. The total sample size for analyses of years ovulating, years of taking the contraceptive pill, and age at last live birth were *N* = 118,227, *N* = 178,233, and *N* = 203,153 respectively. Independent genome-wide significant SNP signals (*p* < 5 × 10^−8^) were identified using the PLINK v1.90b3.31 software package [41]; variants with *r*^2^ > 0.01 with the index SNP and MAF < 0.05 were removed and clump-kb = 1000 for autosomal variants was used to ensure high-quality independent variants were used in the subsequent MR analyses. The previously generated LD reference panel for clumping consisted of a random sample of 47,674 unrelated British UKBB individuals identified using GCTA [40] with identity by state (IBS) < 0.025 and identity by descent (IBD) sharing of < 0.1. LD score regression analysis [42, 43] was used to investigate whether genomic inflation was likely due to polygenicity or population stratification/cryptic relatedness.

### Mendelian randomization analysis

The MR analysis was performed in line with the STROBE-MR checklist [44, 45]. To obtain valid instrumental variables (SNPs) for our analysis, we assessed them against the three core assumptions for MR analysis: (1) That the SNPs are robustly associated with the exposure of interest. For that, we obtained summary result statistics on genome-wide significant SNPs from either our own GWASs or publicly available data to be used in the MR analyses. We assessed instrument strength by calculating the approximate *F* statistic [i.e., *F* ≈ (β/SE)^2^] for the association of the genetic instrument with the exposure and used *F* > 10 to indicate sufficient instrument strength. (2) That the SNPs are not associated with any known or unknown confounders. This is not an assumption that can be fully tested; however, we used PhenoScanner [46, 47] to assess whether any SNPs were associated with known confounders (described below). (3) That the SNPs are not associated with the outcomes through any other path than through the exposure. To test this assumption, we searched PhenoScanner [46, 47] (detailed below) to see if our exposures of interest were associated with other potentially pleiotropic phenotypes. Additionally, we performed MVMR to account for potential pleiotropy. We ran both univariate MR analyses and two sets of MVMR analyses. An overview of the different data sources is given in Table 1.

**Table 1:** Overview of the summary results statistics used in the Mendelian randomization analysis

**Phenotype**		Data source	Study design	Population	Sample size	Phenotype definition	Inclusion/Exclusion criteria	Number of genetic variants	Genetic variant selection	Genetic Variants extracted
**1. BMI**										
BMI [48, 49]	Exposure	UK Biobank and GIANT Consortium	Continous trait	European individuals	434,794	Phenotypes were rank inverse normalized transformed and residualized.	None stated	469	P < 10^−8^	Additional file 1: Table S1.
**2. Ovulatory function**										
Years ovulating*	Exposure	Own GWAS in UKBB	Continous trait	European women	118,227	Years ovulating = years menstruating - years on pill - 0.75 * (live births + stillbirths)	Women with a history of hysterectomy before menopause (N = 12,539), who were not sure of their age at menopause due to their hysterectomy (N = 30,788), or early menopause (< 40 years; N = 3144) were excluded from the analysis.	11	p<5x10^-8^	Additional file 1: Table S2.
Age at Menarche [50, 51]	Exposure	ReproGen Consortium	Continous trait	European women	182,416	Self-reported age at menarche	Women who reported their age at menarche as <9 years or >17 years were excluded from the analysis.	110	p<5x10^-8^	Additional file 1: Table S3.
Age at Menopause [52, 53]	Exposure	ReproGen Consortium	Continous trait	European women	69,360	self-reported and defined as the age at last naturally occurring menstrual period followed by at least 12 consecutive months of amenorrhea.	Included women who were 40–60 years of age, excluding women with menopause induced by hysterectomy, bilateral ovariectomy, radiation or chemotherapy and those using hormone replacement therapy before menopause.	41	p<5x10^-8^	Additional file 1: Table S4.
**3. Reproductive function **										
Number of live births* [54, 55]	Exposure	Own GWAS in UKBB	Continous trait	European women	237,768 women reporting how many children they mothered, 199,570 men reporting how many children they had fathered and 430,466 individuals reporting how many siblings they have.	Number of children mothered adjusted for paternal and child effect.	Participants reporting greater than 10 siblings (N = 1,720, 0.4%) or children (mothered N = 18, 0.007%; fathered N = 43, 0.02%) were recoded to have 10.	6	p<5x10^-8^	Additional file 1: Table S5.
Age at last live birth*	Exposure	Own GWAS in UKBB	Continous trait	European women	220,419	Age at last live birth was defined as the age of primiparous women at birth (for women who reported only one live birth) or the age at last live birth as reported by women who reported multiple live births.	Women with inconsistent reports of age at last live birth across the four data collection instances were excluded (n = 263).	19	p<5x10^-8^	Additional file 1: Table S6.
**4. Oral contraceptive pill **										
We did not identify any genome-wide significant (p<5x10-8) SNPs associated with years taking the oral contraceptive pill, and this phenotype was therefore not taken forward in MR analyses.										
**Outcome**										
Endometrial Cancer [31]	Outcome	ECAC and E2C2 Consortium	Case/Control	European women	12,270 cases and 46,126 controls^a^	Meta-analysis from cohorts using different case inclusion criteria	None stated	(10 as exposure in bidirectional MR)		Additional file 1: Table S7.
	(Exposure in bidirectional MR)									

^*^Exposures considered primary exposures for the univariate Mendelian randomization analysis [31, 48–55]

^a^Excluding UK Biobank. *GWAS*, genome-wide association study; *BMI*, body mass index; *MR*, Mendelian randomization; *ECAC*, Endometrial Cancer Association Consortium; *E2C2*, Epidemiology of Endometrial Cancer Consortium

#### Proxy SNPs

The LDmatrix tool [56] (with the Utah Residents from North and West Europe (CEU) as the reference population) was used to identify high linkage disequilibrium (LD) SNPs to use as proxy SNPs for variants that were missing in any of the exposure or outcome GWASs. An *R*^2^ > 0.8 was required for tag SNPs to be used as proxies.

#### Univariate Mendelian randomization analysis

##### Statistical analysis

We performed a two-sample inverse variance weighted (IVW) MR analysis to assess the causal effect of each exposure on a woman’s risk of EC. To explore potential violations of the MR assumptions, we performed a heterogeneity test using Cochran’s *Q*, and a test for directional pleiotropy was conducted by assessing the degree to which the MR Egger intercept differed from zero [37]. We also performed additional sensitivity analyses using MR Egger regression [37], weighted median [57], and simple and weighted mode estimation methods [58]. Effect estimates from the different sensitivity analysis were compared as a way of assessing the robustness of the results. We acknowledge that these sensitivity analyses may not perform well in some of our analyses where the number of SNPs was low. However, we include the results of these analyses for completeness. Due to the correlation between the exposures in our analysis, we did not perform a strict Bonferroni correction for multiple testing, as this would be too stringent. All univariate MR analyses were performed using the TwoSampleMR package [59] (https://github.com/MRCIEU/TwoSampleMR) in R version 3.5.2 (https://cran.r-project.org/).

##### Investigation of potentially pleiotropic SNPs

SNPs robustly associated with exposures investigated in the MR analyses (number of live births, years ovulating, and age at last live birth) were checked for other possible associations (PhenoScanner v2 [46, 47], http://www.phenoscanner.medschl.cam.ac.uk/) which may contribute to a pleiotropic effect on EC risk. Additional file 1: Table S8 lists the SNPs used in our analysis and shows that many influence more than one exposure, including related phenotypes such as age at menarche and age at menopause. Phenotypes from PhenoScanner were listed in Additional file 1: Table S8 if they were associated with the SNPs or nearby variants in high LD (*r*^2^ = 0.8) at *p*-value level < 1 × 10^–5^ and the phenotype could have a potential pleiotropic effect in the MR analysis. Because of the potential for pleiotropy, MVMR was conducted to tease apart the relationships between the reproductive-related exposures and EC risk.

#### Multivariable Mendelian randomization analysis

##### MVMR phenotype inclusion

Our primary MVMR analysis included the exposures age at menarche, age at menopause, BMI, number of live births, and years ovulating. We chose these phenotypes because univariate MR analyses suggested a causal relationship between each of these exposures and risk of EC. Furthermore, our look-up in PhenoScanner [46, 47], indicated that SNPs that proxied the number of live births and years ovulating were also associated with several phenotypes (i.e., age at menarche, menopause, and/or BMI); therefore, we could use MVMR to account for this potential horizontal pleiotropy. The EC GWAS excluding UKBB individuals was used to extract SNP-outcome associations to be used in the MVMR analysis.

To investigate the potential influence of including the overlapping phenotypes age at menopause and years ovulating, we also ran a secondary MVMR excluding one of these two phenotypes. Lastly, we conducted a third MVMR including the exposures significant in the univariate MR analyses (age at menarche, age at menopause, BMI, number of live births, and years ovulating) in addition to age at last live birth, because of its effect on number of live births in the multivariable observational analysis.

##### MVMR SNP inclusion

Summary statistics for all exposures included in the analysis were all clumped together at once (variants with *r*^2^ > 0.01 with the index SNP were removed) using PLINK [41] as described above. Where pairs of SNPs (associated with different risk factors) were in linkage, variants were preferentially removed from the summary statistics of the phenotype with the largest number of genome-wide SNPs to conserve an adequate number of instruments for each exposure. Only SNPs available across all exposure and outcome summary statistics were included. A full overview of the SNPs included (or reason for exclusion) is listed in Additional file 1: Table S8. Additional univariate MR analyses were performed for each of the exposures, with only the SNPs included in the primary MVMR analysis, to ensure the previously causal relationships were still valid after the clumping procedure.

The MVMR analyses were performed in R (version 4.0.4) using the package MendelianRandomization (version 0.5.0). R code for the univariate and multivariate MR analysis is shown in Additional file 2.

#### Bidirectional Mendelian randomization analysis

We performed bidirectional MR sensitivity analysis between EC risk and number of live births, with EC as the exposure and number of live births as the outcome. Ten SNPs reaching genome-wide significance in the EC GWAS [31] excluding UKBB participants were used as genetic instruments to proxy liability to EC (Additional file 1: Table S7). The effect of the SNP-outcome association was extracted from the maternal specific GWAS summary statistics of the number of live births [54].

## Results

### Observational analyses in UKBB

**Fig. 2:**
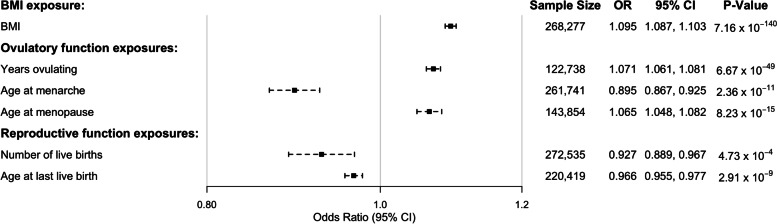
Results from the univariate observational analysis of various risk factors and EC in the UK Biobank. The variables investigated span those related to ovulatory function (age at menarche, age at menopause, and years ovulating), reproductive function (number of live births and age at last live birth), and BMI. Logistic beta and standard errors have been converted to OR and 95% CI. BMI, body mass index; CI, confidence interval; OR, odds ratio

Basic characteristics of the study participants are shown in Additional file 1: Table S9. Significant inverse observational associations were found between EC risk and the number of live births, pregnancy terminations, miscarriages, combined number of live and stillbirths, the total number of pregnancies and age at last live birth, as well as ever taken the oral contraceptive pill, number of years on the oral contraceptive pill, and age at menarche (Fig. 2, Additional file 3: Figure S1; *p* < 0.05). A positive relationship was observed between years ovulating, age at menopause, and BMI and EC risk, whereas we did not find any strong evidence for a relationship between the number of stillbirths and EC risk (Fig. 2, Additional file 3: Figure S1). We also explored the association between EC risk and two possible confounders—Townsend Deprivation Index and educational attainment. Educational attainment was found to be associated with a decreased risk of EC in UKBB women, whereas there was no evidence of association with Townsend Deprivation Index (Additional file 3: Figure S1).

We performed a multivariable regression analysis, where we investigated many risk factors and EC risk simultaneously. The results of these models can be found in Additional file 1: Table S10. When adjusting for age at last live birth, age at menarche, age at menopause, BMI, and educational attainment, the number of live births was no longer significant in our analysis (odds ratio (OR): 1.02, 95% confidence interval (CI): 0.94, 1.10), while the other risk factors remained significant (except for age at menarche). The number of pregnancy terminations showed a strong protective effect on EC risk even when adjusting for the number of live births, age at last live birth, age at menarche, age at menopause, BMI, and educational attainment (OR: 0.82, 95% CI: 0.68, 0.98) (Additional file 1: Table S10). For all the observational analyses, age at last live birth, age at menopause, and BMI remained significant covariates.

A series of sensitivity analyses were conducted to further investigate the number of live birth phenotype, as we found that the number of live births was no longer significant in our multivariate observational analysis when adjusting for other risk factors. We performed a series of regression analyses investigating the effect of number of live births on EC risk, including only one additional risk factor as a covariate at a time (Additional file 1: Table S11). Number of live births remained significantly associated with EC risk in all analyses, except when age at last live birth was added to the model, suggesting it could be age at last live birth, not number of live births that is important in terms of EC risk.

### GWAS analyses in UKBB

We performed genome-wide association analyses on age at last live birth (*N* = 203,153), years taking the oral contraceptive pill (*N* = 178,233), and years ovulating (*N* = 118,227) as there are no previously published GWASs of these traits which identify robustly associated SNPs. We did not find any genome-wide significant SNPs for years taking the oral contraceptive pill, but we did find 11 independent loci associated with age at last live birth and 19 with years ovulating (*p* < 5 × 10^−8^, LD: *r*^2^ < 0.01) (Additional file 1: Table S6 and S2, Manhattan plots are displayed in Additional file 3: Figure S2-S4 and QQ plots in Additional file 3: Figure S5-S7). SNP associations with other traits are shown in Additional file 1: Table S8, suggesting that many of the SNPs for age at last live birth were also associated with educational attainment.

### Mendelian randomization analyses

#### Univariate Mendelian randomization

**Fig. 3:**
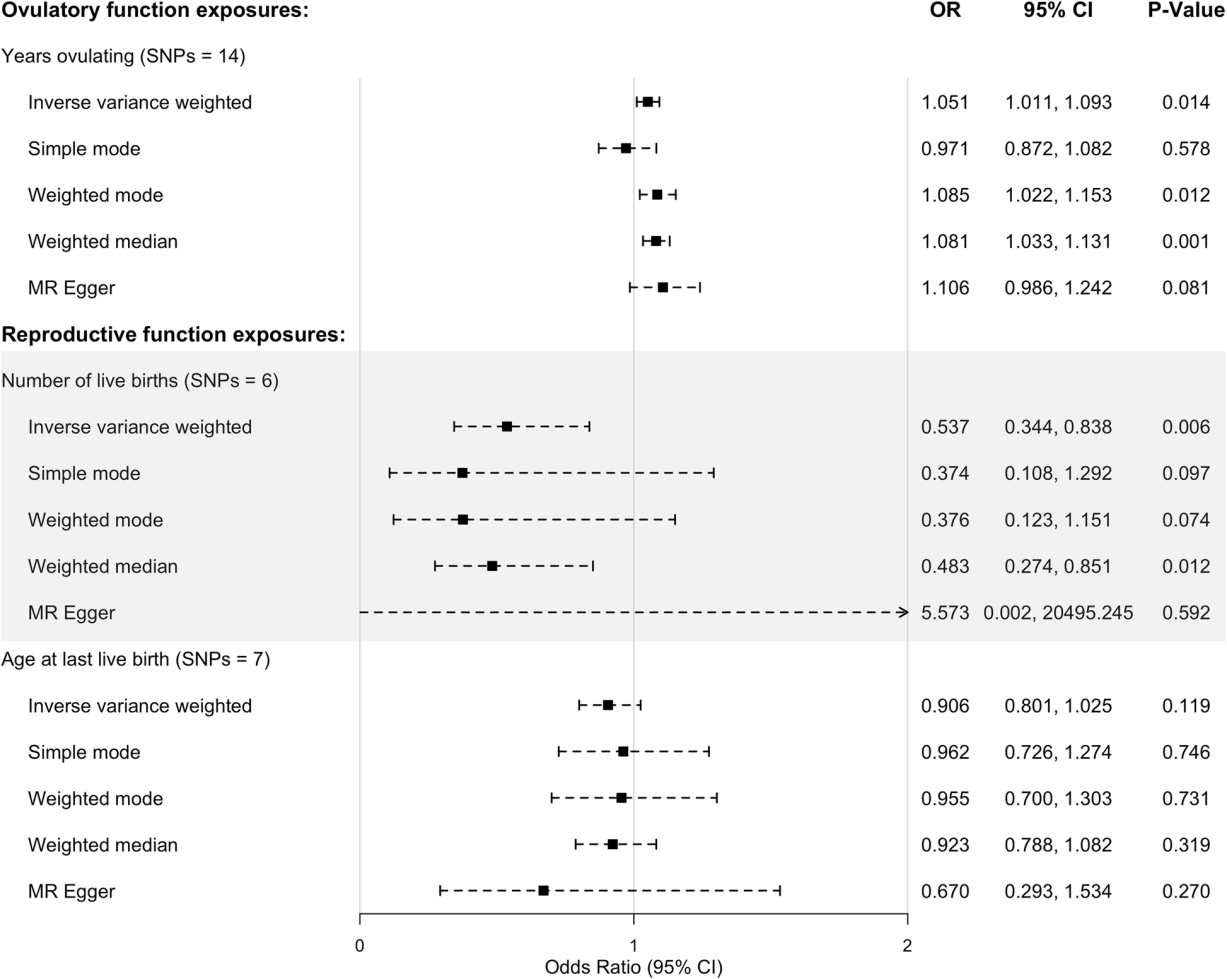
Univariate MR analysis of risk factors of endometrial cancer. All three SNP-exposure betas were obtained from a linear mixed model using fast-GWA for age at last live birth and years ovulating and BOLT-LMM for number of live births [54]. SNP-outcome effect size was log odds from logistic regression. *N*, number; SNP, single nucleotide polymorphism; MR, Mendelian randomization; SE, standard error; CI, confidence intervals

We analyzed the effect of number of live births, age at last live birth, and years ovulating on risk of EC. Since we did not find any loci associated with years on the oral contraceptive pill, we could not include this exposure in the MR analyses. SNPs included in the analyses (or their reason for exclusion) are listed in Additional file 1: Table S8. We found a potential causal relationship between female-specific fertility (number of live births adjusted for paternal and offspring genetic effects) on risk of EC (Fig. 3) in the IVW and weighed median analysis with similar estimates (although with a non-significant *p*-value) for the simple and weighed mode suggesting the number of live births could decrease the risk of EC. The results showed little evidence of heterogeneity (*p*-value 0.630) or directional pleiotropy (MR Egger intercept *p*-value: 0.325; Additional file 1: Table S12). Furthermore, we found a potential causal relationship between increased number of years ovulating and increased risk of EC (Fig. 3), with little evidence of directional pleiotropy (MR Egger intercept *p*-value: 0.318), but some evidence of heterogeneity (*p*-value 0.047) as the simple mode analysis showed opposite direction of effect (Additional file 1: Table S12). We should also note that if we had used a Bonferroni correction in this analysis (0.05/3 = 0.0167), the *p*-value for the IVW years ovulating analysis would only be marginally significant. We found no evidence for a causal effect of age at last live birth on EC risk (Fig. 3). As our lookups in PhenoScanner showed that some of the SNPs associated with age at last live birth and number of live births were associated with potentially pleiotropic phenotypes (Additional file 1: Table S8), we could not conclude regarding causality based on these analyses alone and therefore performed MVMR adding these potentially pleiotropic exposures to the analysis. We used the *F* statistic to inform on instrument strength and found that they all had a high value (> 10) suggesting that the instrument strength is sufficient. Weak instruments can bias the MR causal effect estimate towards the null in two-sample MR analyses, which did not seem to be an issue in our analysis.

#### Multivariable Mendelian randomization

After performing univariate MR analyses, both number of live births and years ovulating showed evidence of a causal relationship with EC. However, look-ups using PhenoScanner (Additional file 1: Table S8) showed that many of the SNPs used to proxy these traits are also associated with other phenotypes thought to causally influence the risk of EC (i.e., age at menarche, menopause, and BMI [31, 32]). We therefore included all these phenotypes in MVMR analyses to account for horizontal pleiotropy that might have affected the univariate MR analyses. After checking LD between the variants associated with the different phenotypes and removing additional SNPs in high LD (i.e., so as to not double count them in the analysis), we reran the univariate MR analyses to check that these exposures still had a significant causal effect on EC risk (Additional file 1: Table S13), and we could not detect any large changes in the results between the full analysis and the analysis only including the SNPs present in the MVMR.

**Fig. 4:**
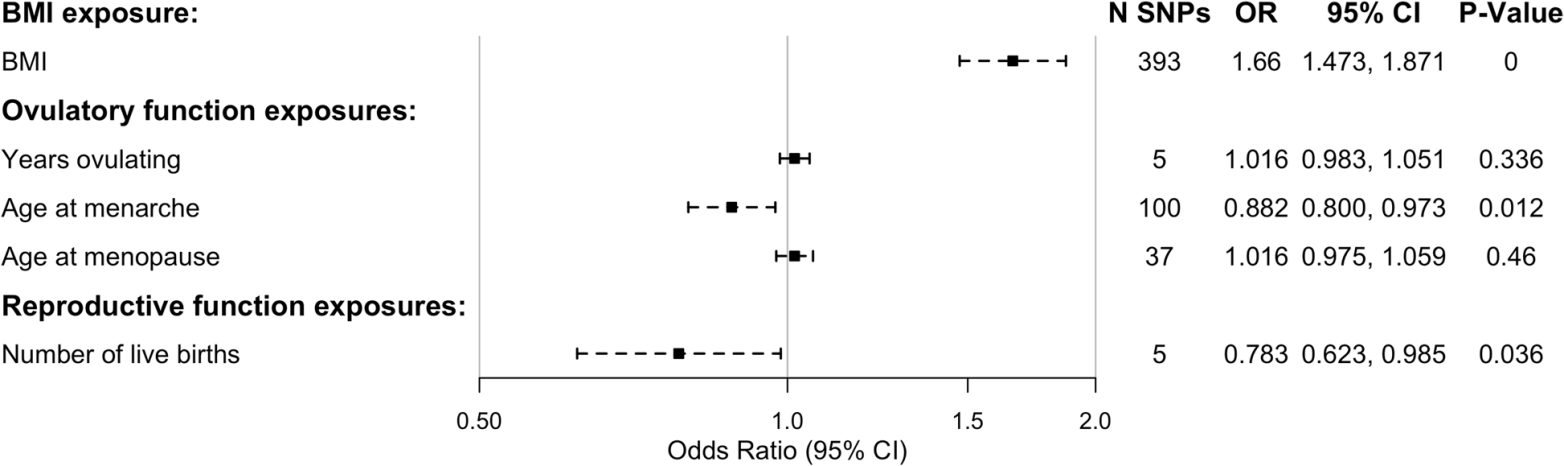
Results from the MVMR analysis of EC risk. All SNP-exposure betas were obtained from linear regression analysis, whereas SNP-outcome effect size was log odds from logistic regression. MVMR, multivariable Mendelian randomization; EC, endometrial cancer; SNP, single nucleotide polymorphism; SE, standard error; BMI, body mass index; CI, confidence interval

Results from the primary MVMR analysis showed an effect of the number of live births, independent of age at menarche, menopause, and BMI (OR: 0.783, 95% CI: 0.623, 0.985); however, visual inspection indicated that the OR attenuated towards 1 compared with the univariate analysis (IVW OR for univariate analysis: 0.537, 95% CI: 0.344, 0.838), suggesting some of the other phenotypes added to the MVMR could have been driving some of the effect observed in those analyses. Years ovulating was not deemed to be an independent causal risk factor for EC (Fig. 4). However, it is important to note that only a small subset of the genome-wide significant SNPs found in the years ovulating GWAS were used in the MVMR due to missingness in some of the other summary statistics. Moreover, many of the years ovulating SNPs were in high LD with genome-wide significant SNPs from the menopause GWAS, and therefore subsequently removed so as not to be counted twice in the analysis. To check if this had any impact on the analysis, we also performed the MVMR analysis excluding either age at menopause or years ovulating from the model. Even when removing one of these variables, the other did not show an association with EC risk independent of age at menarche, number of live births, or BMI (Additional file 1: Table S14). All SNPs included in the MVMR analysis are listed in Additional file 1: Table S15.

Lastly, due to the complicated relationship between the number of live births and age at last live birth observed in the multivariable regression analysis, we performed a third MVMR including age at last live birth as an exposure (see Additional file 1: Table S8 for the additional SNPs included in this analysis). The addition of age at last live birth did not seem to alter the results from the original MVMR (Additional file 1: Table S16).

#### Bidirectional Mendelian randomization analysis

To investigate possible reverse causation between liability to EC and number of live births (i.e., the suggestion that having increased liability to EC would lower an individual’s fertility and therefore number of children), we performed a bidirectional MR analysis with liability to EC as the exposure and number of live births as the outcome. We found that reverse causation was unlikely (*p* > 0.05 across all MR models; Additional file 1: Table S17).

## Discussion

We have performed the largest and most detailed MR analysis of factors related to ovulation and reproductive function and EC risk to date. We found a negative causal effect of the number of live births on a woman’s risk of EC (OR: 0.783, 95% CI: 0.623, 0.985), independent of the causal effect from known risk factors such as age at menarche, age at menopause, and BMI [31–33, 60], which has not been reported to date. These results attenuated compared to the effect of the number of live births on EC risk in the univariate analysis, suggesting some of these other risk factors had a role in this relationship, which could also be the reason some of the sensitivity analyses showed large confidence intervals. Interestingly, we did not find an independent effect of age at menopause, suggesting that this previously reported relationship could be confounded by BMI or age at menarche. Our findings highlight the importance of accounting for other predetermined risk factors with strong effects, when conducting MR analyses.

Additionally, we found a possible causal effect of years ovulating on risk of EC (OR: 1.051, 95% CI: 1.011, 1.093); however, these analyses were only marginally significant and showed some degree of heterogeneity; additionally, the MVMR analysis did not show this to be an independent risk factor after other related factors were taken into account (OR: 1.016, 95% CI: 0.983, 1.051). Indeed, there was a substantial crossover between the SNPs used to proxy the years ovulating and age at menopause variables (which is not surprising given that age at menopause was used to derive the years ovulating phenotype). Consequently, it is likely that our analyses were not well powered to distinguish between these competing explanations of increased EC risk and may be subject to a degree of weak instrument bias when these two exposures were considered in the same multivariate model. MVMR analyses involving at most one of these variables (i.e., years ovulating or age at menopause) suggested that neither exerted strong causal effects on the risk of EC once the other exposures were taken into account.

We have also performed GWAS analyses of the phenotypes age at last live birth, years using the oral contraceptive pill, and number of years ovulating in the UKBB. We found that 11 loci were associated with age at last live birth and 19 loci with years ovulating in post-menopausal UKBB women; however, many of these variants are likely to reflect downstream effects of educational attainment and age at menopause, as confirmed by our look-up using PhenoScanner.

Also, we have shown using univariate and multivariate logistic regression in the UKBB that phenotypes related to reproductive health—such as BMI, years ovulating, and later age at menopause, were all associated with increased risk of EC, whereas educational attainment, age at menarche, contraceptive pill use, age at last live birth, and total number of pregnancies (including pregnancies not going to term) and births (live and still) all show a protective effect on EC risk. This corresponds to previous findings showing that both increased number of full-term pregnancies and miscarriages decrease risk of EC [16, 20]. While the Epidemiology of Endometrial Cancer Consortium [20] used both live and stillbirth to investigate the effect of pregnancy on EC risk, we also investigated this by looking at the effect of both live and stillbirth separately. We found no protective effect of stillbirths in our analysis; however, the confidence intervals were large and further observational analyses with a higher number of cases, as well as further causal analyses, would be beneficial as more data becomes available.

Incomplete pregnancy (defined as miscarriages + induced abortions) has also been associated with decreased EC risk [20], although to a lesser extent when compared to complete pregnancies. Interestingly, we found that number of terminations was associated with a large protective effect on risk of EC. This association has been described before [61, 62] and has been interpreted as evidence that factors early in pregnancy are responsible for the protective effect on EC—in particular, the rapid rise in progesterone to estrogen ratio that occurs in the first few weeks after conception [62]. However, our analyses showed a substantially stronger protective effect of the number of terminations compared to the number of miscarriages or even the number of live births. This relationship between the number of terminations and EC risk remained strong in our multivariable regression analysis adjusting for number of live births, age at last live birth, age at menarche, age at menopause, BMI, and educational attainment. A recent Danish study reported similar findings [62]. It is unclear what is responsible for this difference in risks between miscarriage and terminations. It could be, for example, that factors later in pregnancy offset some of these reductions in risk. However, our results show that number of miscarriages (which is defined as pregnancy loss before week 24 in the UK (up until 1991 defined as pregnancy loss before week 28)) has a similar effect size as number of live births arguing against this possibility. Alternatively, it may be that factors specific to the termination process have a stronger protective effect (e.g., surgical abortion or vacuum aspiration removes neoplastic cells) than those related to the miscarriage process, or that women whom have terminations systematically differ from those who do not.

### Strengths and limitations

One of the strengths of our study is the ability to use MVMR analysis to explore the effect of the number of live births conditional on correlated phenotypes such as age at menarche, menopause, and BMI. While we did perform a series of sensitivity analyses to explore potential bias that might arise in univariate MR analyses due to horizontal pleiotropy, we acknowledge that these methods work best with more genetic instruments than we had available in our analyses. MVMR can account for such horizontal pleiotropy acting on the outcome through the modeled exposures and therefore has allowed estimation of direct causal effects of each exposure on EC risk.

Unfortunately, we did not find any valid genetic instruments for oral contraceptive pill use and were unable to perform any MR analyses of the causal effect of contraceptive pill use on the risk of EC. Larger GWAS analyses could make these analyses possible in the future; however, it could also be that this variable is largely affected by non-genetic factors, and that it therefore might not be possible to perform MR analysis on this trait in general. Another issue with this phenotype is that in the UKBB there is no information on the type of oral contraceptive pill. There might for instance be different effects of pill use on EC risk depending on the hormone combination and dosage [63]. For example, oral contraceptives widely used in the 1960s generally contained higher doses of estrogen, with up to 150 µg estrogen compared to the present dosage of 20 to 30 µg [14]. It is not known from observational studies, whether this reduction in estrogen and changes between different generations of progestins influence EC risk reduction. Contraceptive pill use is also a crude measurement as we only have age of first and last use in the UKBB, and many women would have stopped taking the pill at some stage during their lifetime to have children.

However, various mechanisms for this observational association between oral contraceptive pill use and the reduction in EC risk have been proposed, including that suppressing endometrial cell proliferation via a reduced number of ovulations is beneficial. For instance, reduced lifetime number of ovulatory cycles and years menstruating have been associated with a reduction in EC risk [4, 5]. To capitalize on the information available in the UKBB, we created the variable years ovulating to try to test if this observational relationship is causal. Unfortunately, many of the genetic variants associated with the trait were either unavailable in the other summary statistics used in the MVMR or also strongly associated with age at menopause, leaving only a few instruments available for the MVMR analysis. Larger GWASs for these traits could potentially inform more precisely on the effect of the oral contraceptive pill and ovulation on EC in the future.

Another potential issue is that lifetime ovulation cycles [4], or lifetime number of years of menstruation [5], did not account for time spent breastfeeding or variation in the length of an individual’s ovulation cycle. Data on breastfeeding duration is unfortunately not available in the UKBB and length of ovulation cycle is only available in a small subsample of women. GWAS analyses performed including these variables might yield better instruments for use in further MR analyses. We also acknowledge that the age for the UKBB women may be lower than the peak incidence age for EC, which could influence the results of the observational analysis.

Likewise, there are not enough genetic instruments associated with either miscarriages, stillbirths, or pregnancy terminations to run MR analysis and we therefore could not include these potentially causal phenotypes in our MVMR analysis. Our observational analysis suggests these phenotypes might have a protective effect on EC and future studies should aim to investigate this further.

Notably, there appears to be an inconsistency between the observational analyses and the MR analyses. While the univariate observational analyses suggest that number of live births is negatively associated with EC risk, the multivariate observational analyses (Additional file 1: Table S10) show an attenuation of the association between the number of live births and EC risk when accounting for other risk factors, in particular age at last live birth (Additional file 1: Table S11). Age at last live birth is positively correlated with the number of live births, and both risk factors are negatively associated with EC risk in the univariate observational analyses. However, our univariate MR analyses found that the number of live births, and not age at last live birth, is causally related to EC risk. Likewise, the MVMR analysis of the exposures age at menarche, age at menopause, years ovulating, number of live births, and age at last live birth found that the number of live births and not age at last live birth is causally related to EC risk (Additional file 1: Table S16). The null result for age at last live birth in the MR analyses could be due to the lack of strong genetic instrumental variables, or the inconsistency could simply be the result of confounding. Nevertheless, these puzzling results should be further investigated in independent cohorts when more genetic instrumental variables for each exposure are available.

The inability to find a causal effect of age at menopause on EC risk in the MVMR analysis could be because of HRT. HRT is used to ameliorate menopausal symptoms, and if estrogen only HRT is administrated it is associated with increased EC risk [8]. Women who experienced menopause at a younger age—which theoretically should decrease the risk of EC—could increase their risk of EC by going on this form of HRT. Unfortunately, no information regarding the type of HRT used was available for UKBB women to be added to the analysis to try to answer this question.

Additionally, MR analysis has shown a causal link between estradiol and EC [9] whereas we found that number of terminations was associated with a large protective effect on the risk of EC in our observational analysis, which could be due to the rapid rise in progesterone to estrogen ratio that occurs in the first few weeks after conception [62]. Progesterone is known to downregulate estrogen receptors in the endometrium and promote cell differentiation, thus opposing the mitogenic effects of estrogen [10, 11]. Our analysis suggests that it may not be estrogen itself that is causal for the development of EC, but the estrogen/progesterone ratio, something we could not analyze in our MR analysis as not genetic variants for this ratio is available.

We also acknowledge that we were unable to adjust for age at EC diagnosis in our analysis, as this information was not available to us. As age is an important risk factor, this is a significant limitation to our observational analysis. Additionally, we do acknowledge that our study population consists of women with European ancestry, which is a limitation to our study. In addition, there could be a selection bias within the UKBB, which could lead to bias in our observational analysis. More research is needed on diverse populations before strong conclusions can be made.

Taken together, both the observational and causal results suggest that having been pregnant has a protective effect on the risk of EC. Our analysis is consistent with findings from observational studies—where a leading hypothesis has been that a decrease in exposure to fluctuating sex hormones (either because of time spent on the oral contraceptive pill [13] or time spent pregnant [16–20], having early menopause [3] or late menstruation [3]) could be protective for EC.

## Conclusions

In conclusion, we have triangulated various traditional and novel approaches to investigate the relationship between various risk factors and EC risk in the UKBB. We found evidence that BMI, age at menarche, and the number of live births each had independent causal effects on EC risk. To our knowledge, this is the first study to report that number of live births may have a protective effect on the risk of EC, even when accounting for other risk factors. However, our analyses suggest that the causal effect of the number of live births on EC risk may be driven through the age when a woman last gave birth, and this relationship should be further investigated. Our observational analyses suggested strong effects for years ovulating and contraceptive pill use on EC risk; however, we were not able to replicate that in the MR analysis.

## Supplementary Information

**Additional file 1: Table S1.** Genome-wide significant independent SNPs for Body Mass Index in women (Pulit 2018). **Table S2.** Genome-wide significant independent SNPs for Years Ovulating (Hg19). **Table S3.** Genome-wide significant independent SNPs for age at menarche downloaded from the ReproGen Consortium Website (Perry et al., 2014; Hg19). **Table S4.** Genome-wide significant independent SNPs for age at menopause downloaded from the ReproGen Consortium Website (Hg19; Day et al., 2015). **Table S5.** Genome-wide significant independent SNPs for number of live births extracted from Warrington et al., 2021 (Hg19). **TableS6.** Genome-wide significant independent SNPs for age at last live birth (Hg19). **Table S7.** Genome-wide significant independent SNPs for Endometrial Cancer used in the bidirectional MR analyses (Hg19). **Table S8.** Overview of SNPs and possible pleiotropic effects (Hg19). The direction of the association with other traits in PhenoScanner (i.e. positive (+) or negative(-)) is indicated. **Table S9.** Basic characteristics of the study participants in UK Biobank. **Table S10.** Results from the multivariate observational analyses of reproductive risk factors and endometrial cancer risk. **Table S11.** Results from the multivariate observational analyses of number of live births and other reproductive risk factors on endometrial cancer risk. **Table S12.** Heterogeneity and Directional Pleiotropy tests from MR analysis of female fertility and risk of endometrial cancer. **Table S13.** Univariate Mendelian randomization results with only the SNPs included in the primary multivariable MR. **Table S14.** Results from two Multivariable Mendelian Randomization analyses including the exposures A) BMI, age at menarche, age at menopause and number of live births; and B) BMI, age at menarche, years ovulating and number of live births. **Table S15.** Overview of all SNPs (Hg19) and their association with the different traits included in the multivariable MR analysis. **Table S16.** Results from the Multivariable Mendelian Randomization analysis including the exposures age at menarche, age at menopause, years ovulating, number of live births and age at last live birth. **Table S17.** MR analysis of liability to endometrial cancer on number of live births.

**Additional file 2.**

**Additional file 3.**

## Data Availability

UK Biobank (https://www.ukbiobank.ac.uk/) data is available to researchers upon application to the individual cohorts via their websites. All other data used are publicly available and referenced according in the main text. GWAS summary results of statistics for years ovulating, age at last live birth, and years taking the oral contraceptive pill are available through the following link: https://cloudstor.aarnet.edu.au/plus/s/RkRnbpX5WPU3QI3.

## Abbreviations

BMI: Body mass index
CEU: Utah Residents from North and West Europe
CI: Confidence interval
EC: Endometrial cancer
GCTA: Genome-wide Complex Trait Analysis
GRM: Genetic relationship matrix
GWAS: Genome-wide association study
HRT: Hormone replacement therapy
IBD: Identity by descent
IBS: Identity by state
IVW: Inverse variance weighted
LD: Linkage disequilibrium
MAF: Minor allele frequency
MR: Mendelian randomization
MREC: Multi-Centre Research Ethics Committee
MVMR: Multivariable Mendelian randomization
OR: Odds ratio
SNP: Single nucleotide polymorphism
UKBB: UK Biobank
