# Supplementary material for: Mendelian randomization analysis of factors related to ovulation and reproductive function and endometrial cancer risk
Source: BMC Med. 2022 Nov 1;20:419. doi: 10.1186/s12916-022-02585-w (PMC9623961; doi:10.1186/s12916-022-02585-w)
Supplement: Supplementary file 2 — Additional file 2. [file 12916_2022_2585_MOESM2_ESM.docx]

# Supplementary R Code

# Load libraries

library(data.table)

library(MendelianRandomization)

library(TwoSampleMR)

##########################################################################

# 1. Sample code for the univariate mendelian randomisation (MR) analyses

##########################################################################

# Read in the summary statistics for the exposure (eg. years ovulating)

exp_dat <- read_exposure_data("YO_EXPOSURE_DATA.txt", sep="\t",

snp_col = "SNP", beta_col = "BETA",

se_col = "SE", effect_allele_col = "EA",

other_allele_col = "NEA", pval_col = "P", eaf_col = "AF_EA")

# Specify the exposure name

exp_dat$exposure = "Years ovulating"

# Read in the summary statistics for the outcome (eg. endometrial cancer)

out_dat <- read_outcome_data(filename = "YO_OUTCOME_DATA.txt",

sep = "\t", snps = exp_dat$SNP,

snp_col = "SNP", beta_col = "BETA",

se_col = "SE", effect_allele_col = "EA", other_allele_col = "NEA",

eaf_col = "EAF", pval_col = "P")

# Specify the outcome name

out_dat$outcome = "Endometrial cancer"

# Harmonise the data

data <- harmonise_data(exp_dat, out_dat, action = 2)

# Run the univaraite MR analyses using various MR approaches

mr_results <- mr(dat, method_list=c("mr_egger_regression", "mr_ivw", "mr_weighted_median", "mr_simple_mode", "mr_weighted_mode"))

# Create a scatter plot

mr_scatter_plot(mr_results, dat)

# Heterogeneity test

mr_heterogeneity(data)

# Pleiotropy test

mr_pleiotropy_test(data)

# Single SNP approach

res_single <- mr_singlesnp(data)

# Forest plot of the single SNP approach

mr_forest_plot(res_single)

# Funnel plot of the single SNP approach

mr_funnel_plot(res_single)

##########################################################################

# 2. Sample code for the multivariable MR analyses

##########################################################################

# Read in file containing merged summary statistics (outcome and exposures)

SUMM_STATS <- fread("SUMM_STATS.txt", header = T)

# Note that you should check that the SNPs are aligned (ie. same effect alleles: EA)

# If not, adapt the below code (for each exposure)

# Create a new column for aligned beta values and fill with NAs

#DATA$BETA_new<- NA

# For each SNP

# If EA_outcome = EA_exposure, do not change the beta

# if EAs differ, swap the sign of the exposure beta

#for(i in 1:dim(DATA)[1]) {

# DATA$BETA_new[i] <- if(DATA$EA_outcome[i] == DATA$EA_exposure[i]){

# DATA$BETA_exposure[i]

# } else {

# - DATA$BETA_exposure[i]

# }

#}

# Set up the multivariable MR input object

# Beta = effect size, SE = standard error

# Exposures: MENA = age at menarche, MENO = age at menopause

# YO = years ovulating, BMI = body mass index

# NLB = number of live births

# Outcome: EC = endometrial cancer

MRMVInputObject <- mr_mvinput(bx = cbind(SUMM_STATS$Beta_MENA,

SUMM_STATS$Beta_MENO, SUMM_STATS$BETA_YO, SUMM_STATS$Beta_BMI, SUMM_STATS$Beta_NLB),

bxse = cbind(SUMM_STATS$SE_MENA,

SUMM_STATS$SE_MENO, SUMM_STATS$SE_YO, SUMM_STATS$SE_BMI, SUMM_STATS$SE_NLB),

by = SUMM_STATS$Beta_EC,

byse = SUMM_STATS$SE_EC)

# Run the multivariable MR analysis

MRMVObject_proxy <- mr_mvivw(MRMVInputObject, model = "default", correl = FALSE, distribution = "normal", alpha = 0.05)
