# Supplementary material for: Mendelian randomization analysis of factors related to ovulation and reproductive function and endometrial cancer risk
Source: BMC Med. 2022 Nov 1;20:419. doi: 10.1186/s12916-022-02585-w (PMC9623961; doi:10.1186/s12916-022-02585-w)
Supplement: Supplementary file 3 — Additional file 3. [file 12916_2022_2585_MOESM3_ESM.docx]

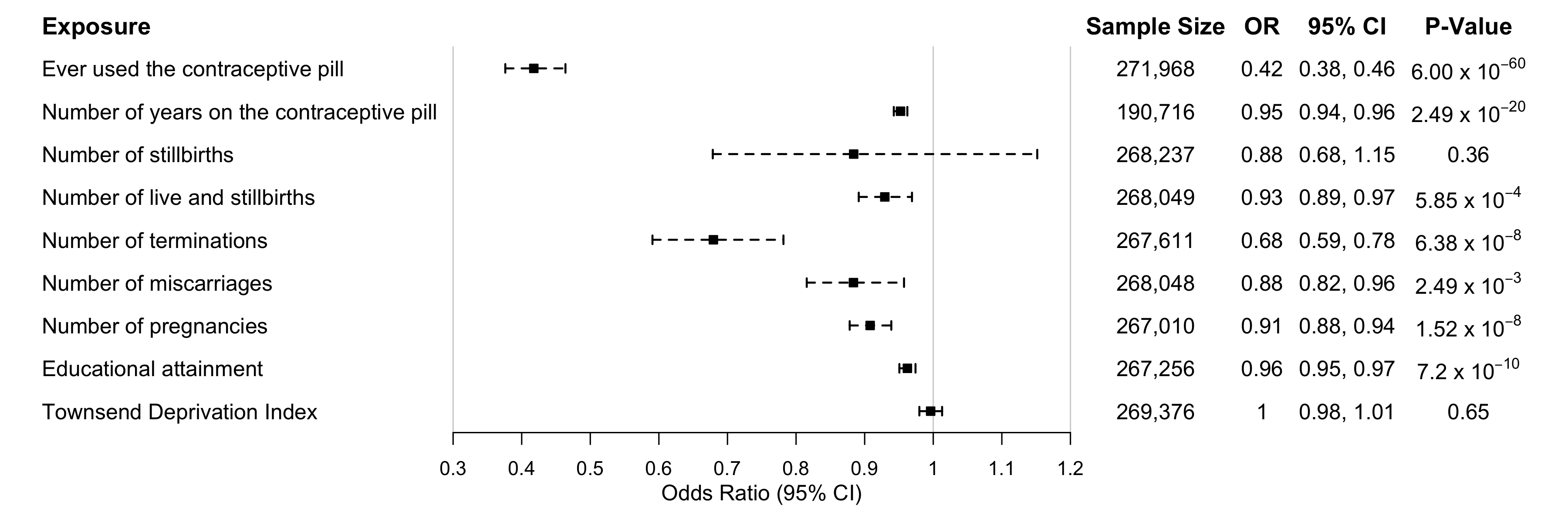


**Figure S1. Results from the univariate observational analysis of additional reproductive factors of interest and risk of EC in the UK Biobank.** Logistic beta and standard errors have been converted to OR and 95% CI. OR: Odds Ration; CI: Confidence interval.


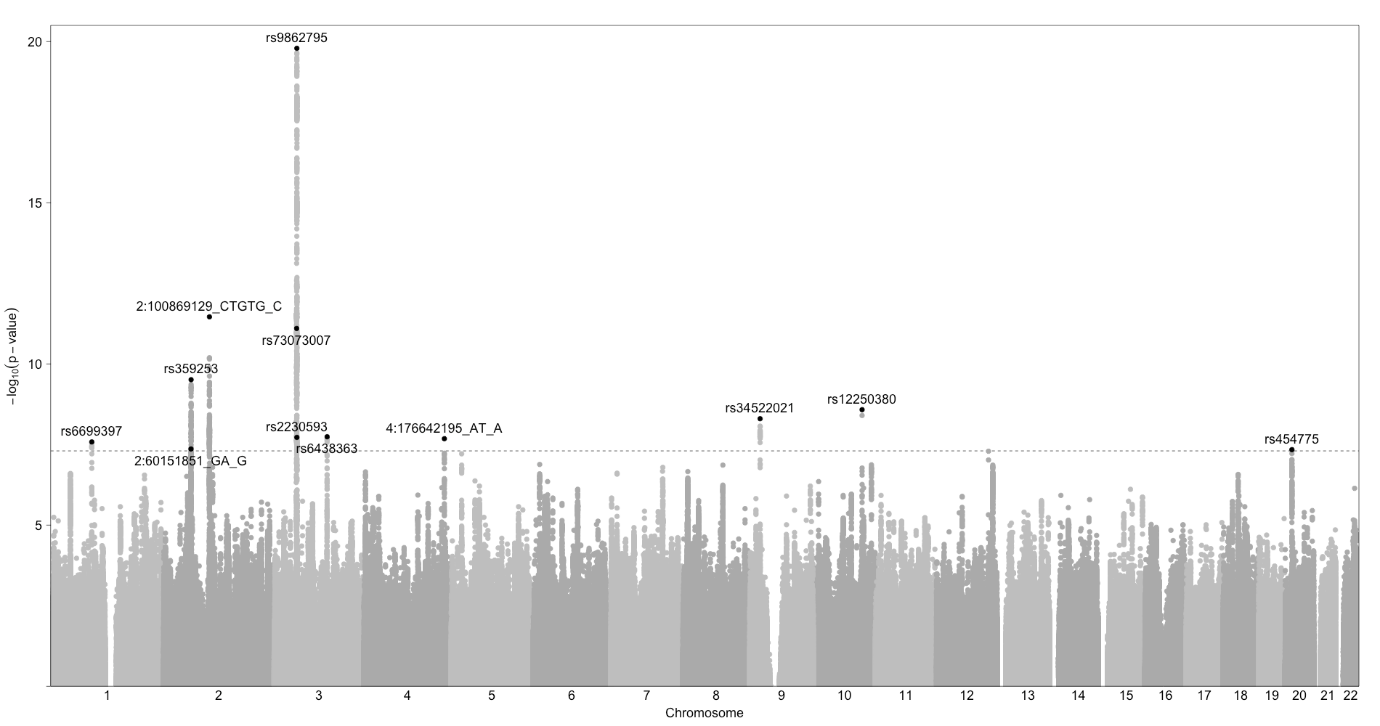


**Figure S2: Manhattan plot of age at last live birth.** Each dot represents a genetic variant. The X-axis shows the position of the genetic variants on the chromosomes and the Y-axis displays the –log10 p-value. The dashed line represents genome-wide (p = 5 x 10^-8^).


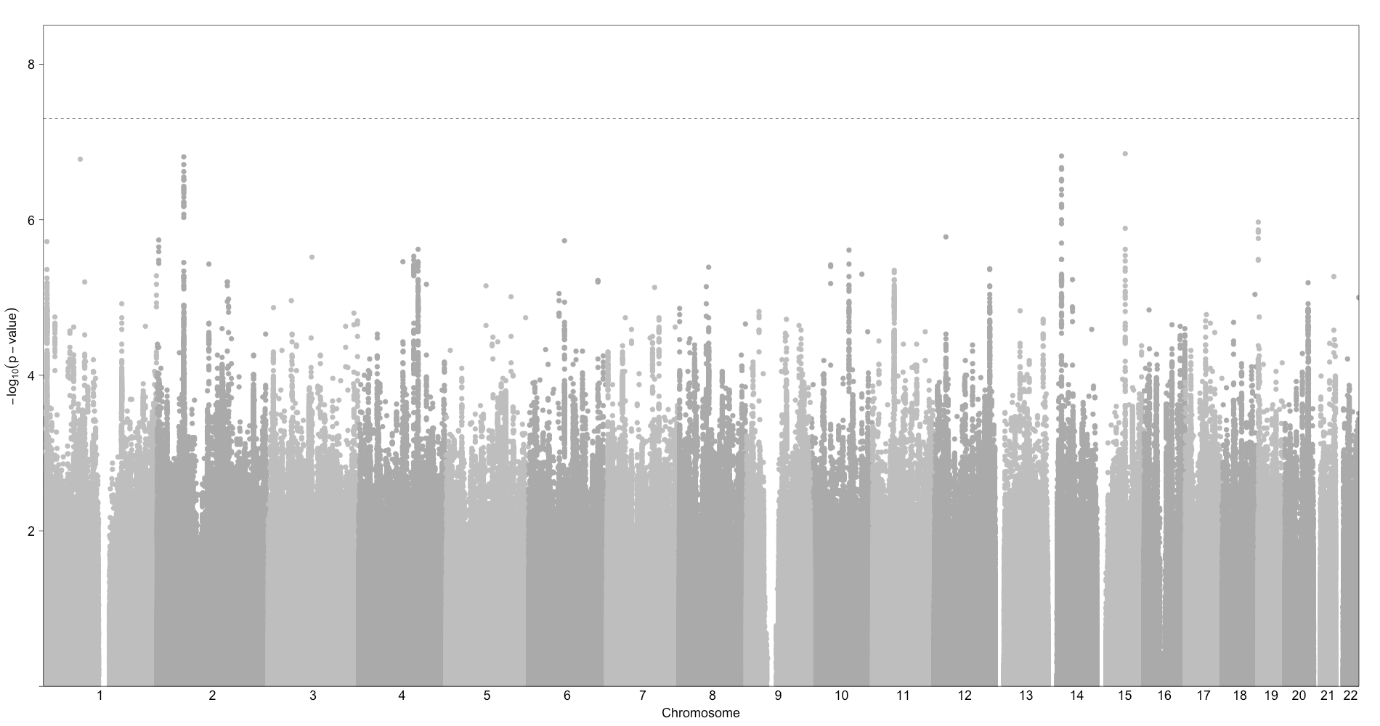


**Figure S3: Manhattan plot of years taking the oral contraceptive pill.** Each dot represents a genetic variant. The X-axis shows the position of the genetic variants on the chromosomes and the Y-axis displays the –log10 p-value. The dashed line represents genome-wide (p = 5 x 10^-8^).


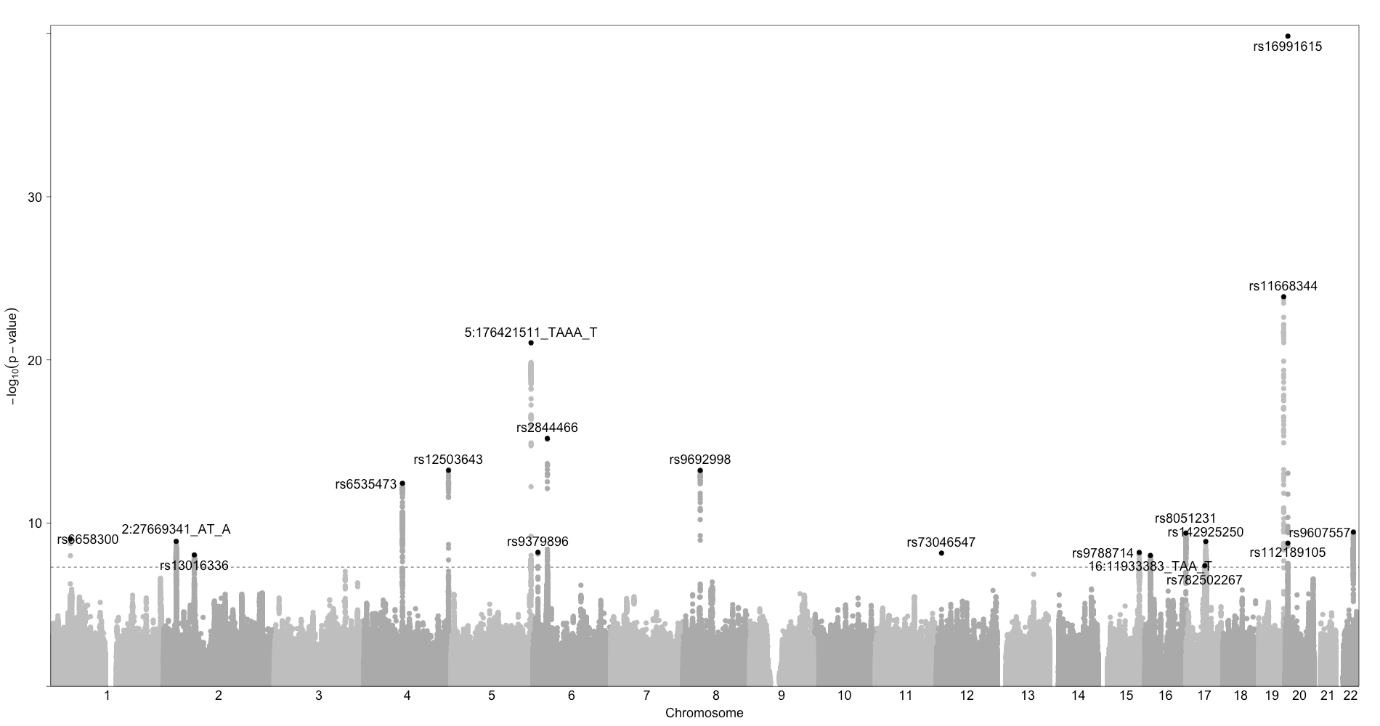


**Figure S4: Manhattan plot of years ovulating.** Each dot represents a genetic variant. The X-axis shows the position of the genetic variants on the chromosomes and the Y-axis displays the –log10 p-value. The dashed line represents genome-wide (p = 5 x 10^-8^).


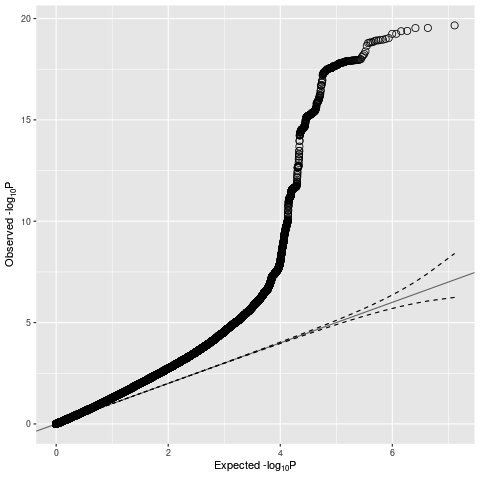


**Figure S5: Quantile-quantile plot for the GWAS of age at last live birth comparing the observed GWAS p-values to those expected under a null distribution**. The mixed linear model GWAS analysis for age at last live birth included UKBB assessment center, genotyping batch, year of birth and the top ten genetic principal components as covariates. The dotted lines indicate the 95% confidence intervals. There is some evidence of inflation, indicated by early departure of observed p-values from the middle diagonal line. Lambda = 1.38; LD score regression intercept: 1.02.


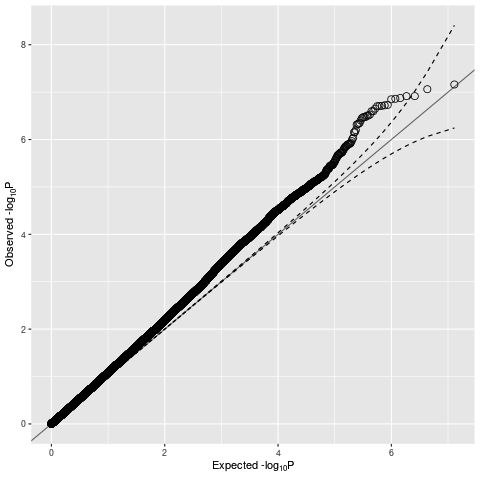


**Figure S6: Quantile-quantile plot for the GWAS of years taking the oral contraceptive pill comparing the observed GWAS p-values to those expected under a null distribution**. The mixed linear model GWAS analysis for years taking the oral contraceptive pill included UKBB assessment center, genotyping batch, year of birth and the top ten genetic principal components as covariates. The dotted lines indicate the 95% confidence intervals. There is some evidence of inflation, indicated by early departure of observed p-values from the middle diagonal line. Lambda = 1.09; LD score regression intercept: 1.00.


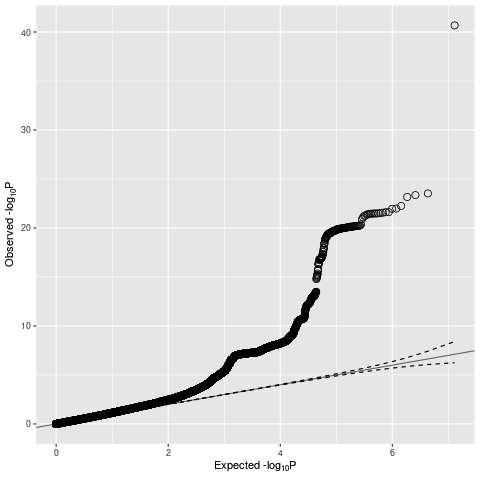


**Figure S7: Quantile-quantile plot for the GWAS of years ovulating comparing the observed GWAS p-values to those expected under a null distribution**. The mixed linear model GWAS analysis for years ovulating included UKBB assessment center, genotyping batch, year of birth and the top ten genetic principal components as covariates. The dotted lines indicate the 95% confidence intervals. There is some evidence of inflation, indicated by early departure of observed p-values from the middle diagonal line. Lambda = 1.15; LD score regression intercept: 0.99.
